# Precision oncology: Artificial intelligence, circulating cell‐free DNA, and the minimally invasive detection of pancreatic cancer—A pilot study

**DOI:** 10.1002/cam4.6604

**Published:** 2023-10-03

**Authors:** Ray O. Bahado‐Singh, Onur Turkoglu, Buket Aydas, Sangeetha Vishweswaraiah

**Affiliations:** ^1^ Department of Obstetrics and Gynecology Corewell Health – William Beaumont University Hospital Royal Oak Michigan USA; ^2^ Department of Care Management Analytics Blue Cross Blue Shield of Michigan Detroit Michigan USA; ^3^ Department of Obstetrics and Gynecology Corewell Health Research Institute Royal Oak Michigan USA

**Keywords:** artificial intelligence, circulating cell‐free DNA, DNA methylation, epigenetics, pancreatic cancer, precision oncology

## Abstract

**Background:**

Pancreatic cancer (PC) is among the most lethal cancers. The lack of effective tools for early detection results in late tumor detection and, consequently, high mortality rate. Precision oncology aims to develop targeted individual treatments based on advanced computational approaches of omics data. Biomarkers, such as global alteration of cytosine (CpG) methylation, can be pivotal for these objectives. In this study, we performed DNA methylation profiling of pancreatic cancer patients using circulating cell‐free DNA (cfDNA) and artificial intelligence (AI) including Deep Learning (DL) for minimally invasive detection to elucidate the epigenetic pathogenesis of PC.

**Methods:**

The Illumina Infinium HD Assay was used for genome‐wide DNA methylation profiling of cfDNA in treatment‐naïve patients. Six AI algorithms were used to determine PC detection accuracy based on cytosine (CpG) methylation markers. Additional strategies for minimizing overfitting were employed. The molecular pathogenesis was interrogated using enrichment analysis.

**Results:**

In total, we identified 4556 significantly differentially methylated CpGs (*q*‐value < 0.05; Bonferroni correction) in PC versus controls. Highly accurate PC detection was achieved with all 6 AI platforms (Area under the receiver operator characteristics curve [0.90–1.00]). For example, DL achieved AUC (95% CI): 1.00 (0.95–1.00), with a sensitivity and specificity of 100%. A separate modeling approach based on logistic regression‐based yielded an AUC (95% CI) 1.0 (1.0–1.0) with a sensitivity and specificity of 100% for PC detection. The top four biological pathways that were epigenetically altered in PC and are known to be linked with cancer are discussed.

**Conclusion:**

Using a minimally invasive approach, AI, and epigenetic analysis of circulating cfDNA, high predictive accuracy for PC was achieved. From a clinical perspective, our findings suggest that that early detection leading to improved overall survival may be achievable in the future.

## 1 INTRODUCTION

Pancreatic cancer (PC) is a lethal malignancy^1^ and is predicted to become the second leading cause of cancer deaths in the US by 2030.^2^ The principal cause of this poor prognosis is the late presentation, a direct consequence of the lack of early screening markers. Late clinical presentation is characterized by disseminated spread and resistance to chemotherapy.^2^ The ultimate objective of precision medicine (PM) is the development of targeted individual therapy. This will in part be accomplished using approaches such as genomics and advanced computational tools. Biomarker development is critical to PM. The primary application of PM has been precision oncology.^3^ Since its establishment as an NIH priority area, there has been a global explosion in publications related to precision oncology.^4^ Indeed, the concept of PM has now been seeded in other unrelated disciplines.^5^

Circulating cell‐free DNA (cfDNA) refers to DNA that is present in the bloodstream and that exists outside of the cells.^6^ Recent publications have suggested the value of epigenomic analysis for the elucidation of cancer pathogenesis and for accurate minimally invasive detection^7^, ^8^ using cfDNA. Methylation markers identified using cfDNA have the potential to function as a standalone predictor of progression‐free survival,^9^ and treatment response^10^ in PC. Recent studies have shown that cfDNA from patients with PC contains unique methylation patterns that are specific to the disease and can provide valuable information on PC biology.^11^ Based on the above, we evaluated the use of circulating cfDNA methylation analysis for the accurate prediction of PC and for investigating PC pathobiology.

Artificial intelligence (AI) refers to the ability of computers to perform tasks that were once considered uniquely human, such as reasoning and learning. AI is a branch of computer science where machines can synthesize data presented to them, learn patterns therefrom, and identify these patterns and associations in new datasets. An exciting application of AI is the ability to identify previously unrecognized, defining features in a dataset and accurately classify or distinguish groups. In the biological sciences, AI can now be coupled with genomics for highly accurate disease detection and to elucidate the mechanisms of complex disorders including cancer.^7^, ^8^ In the era of multi‐omics studies, the capability of AI adds significantly to the analysis and interpretation of omics big data. In the present study, we sought to combine DNA methylation analysis of circulating cfDNA with AI analysis to identify minimally invasive biomarkers and to investigate the epigenomic pathogenesis of PC.

## 2 MATERIALS AND METHODS

The Institutional Review Board of Beaumont Health, Royal Oak, MI, approved the study protocol (IRB#2018‐306). Participants provided written consent. Blood was obtained from seven treatment‐naïve PC patients and with 14 controls who had no diagnosis or suspicion of cancer. All the PC cases had histological confirmation of the diagnosis, and none received radiation, chemotherapy, or surgical therapy prior to sample collection. The specimens were collected in Streck Cell‐Free DNA BCT® tubes,^12^ storage conditions,^13^ and cfDNA processing methods using QIAamp circulating nucleic acid kit (Qiagen Cat # 55114) were described in a prior publication.^7^ DNA methylation profiling was performed using the EZ DNA Methylation Kit (Zymo) and^14^ and the Illumina Infinium MethylationEPIC BeadChip arrays as per manufacturer's instructions (Illumina, Inc.).

### 2.1 Statistical analysis

Raw iDAT files of Illumina EPIC array data were processed using the R package (v 4.1.1). The package's “minfi” and “noob” normalization method was used for data normalization. Outlier detection was performed, and two control samples were considered as outliers^15^ and removed from further analysis. Cell type deconvolution was performed with blood immune cell types as the reference population. None of the estimated cellular populations showed a significant difference between the groups. After analysis of variance inflation, cfDNA contribution from hemolyzed leukocytes namely CD4T, CD8T, and Granulocytes was found to be inflating the data and was removed from further analysis. We retained age, sex, B‐cell, monocytes, and natural killer cells as covariates in subsequent linear regression models. The “limma” package was used to determine differentially methylated cytosines. All cytosine CpGs were annotated with genomic and island regions followed by their enrichment was estimated using Fisher's exact test. Analysis details are as described in prior publications.^7^, ^8^

### 2.2 Gene enrichment analysis

A graphical enrichment analysis tool “ShinyGO 0.77” was used to perform the gene enrichment analysis. Enrichment of pathways was performed using the previously described approaches.^16^, ^17^, ^18^

### 2.3 Imprinted gene analysis

We performed a search using “geneimprint” database (https://www.geneimprint.com/site/genes‐by‐species) to help elucidate the role of imprinted genes in PC pathogenesis.

### 2.4 Artificial intelligence analysis

Comprehensive AI analyses to identify the optimal CpG markers for distinguishing the groups and predicting PC after normalization procedures, detailed in a prior publication,^19^ were performed. A total of six AI algorithms or platforms: Deep Learning (DL), Support Vector Machine (SVM), Generalized Linear Model (GLM), Prediction Analysis for Microarrays (PAM), Random Forest (RF), and Linear Discriminant Analysis (LDA), were employed for both classification and regression analysis.^20^ A brief description of each of these platforms was previously reported^21^ and is briefly summarized in Data S1. We separately determined the predictive accuracy of CpGs within gene regions (intragenic CpGs) and those outside gene regions (extragenic CpGs). The method of modeling and evaluation that was utilized in this study involved a two‐step validation approach using two different data sets. Two techniques were used to find the best model and calculate performance metrics: 5‐fold cross‐validation and bootstrapping. With 5‐fold cross‐validation, the dataset was randomly divided into a training and a test set. The model was fitted to the training set and tested in the independent test set. This process was repeated 10 times, and the results were averaged to obtain the performance metrics. In the bootstrapping technique, new data sets were generated by repeatedly sampling observations from the original dataset with replacement. Each of these “bootstrap datasets” was used as a training sample, and the original dataset was used as a test sample. This process was also repeated 10 times, and the results were averaged to obtain performance metrics. Both techniques were used for validation on a separate validation dataset, and the results were provided separately for the validation group. The detailed methods of training and validating the data are provided in Data S1.

### 2.5 Minimizing overfitting

Due to the relatively small sample size, there is a risk of overfitting. We minimized overfitting using the following strategies. For the DL model, we applied L1 and L2 regularization parameters, causing some weights to become 0 and preventing weight enlargement. Additionally, we utilized the “input dropout ratio” to control overfitting with respect to high‐dimensional noisy data. For other AI platforms, we tuned various parameters, such as number of trees for RF, classification cost for SVM, and threshold amount for PAM, to overcome the challenge of overfitting.

### 2.6 Multivariate analysis and regression models

Multivariate analysis including partial least squares discriminant analysis (PLS‐DA) and logistic regression models were performed using the R package through MetaboAnalyst (v 5.0).^22^ Prior to performing PLS‐DA, all data were normalized to the median and auto‐scaled.^23^ Models were cross‐validated using permutation testing (2000 iterations) to determine whether the observed separation in the representative scores' plots achieved statistical significance. Subsequently, logistic regression analysis was performed using a stepwise variable selection of CpGs to optimize all the model components. A k‐fold cross‐validation (CV) technique ensures the validity and generalizability of our logistic regression model by randomly dividing the entire sample data into “k” equal‐sized subsets. Optimal and robust predictive algorithms were generated.^24^ The predictive accuracy of regression models was determined based on the calculation of the area under the receiver operating characteristics curve (AUROC or AUC), sensitivity, and specificity values.

## 3 RESULTS

There were a total of seven treatment‐naïve PC cases and 12 unaffected controls analyzed after elimination of two outliers. The detected outliers are pictorially represented in Figure S1. Variance inflation in the study data is presented in Figure S2. Clinical and demographic data are shown in Table S1 along with the details of the PCs histology. There were no significant differences in age, BMI, or gender between groups. Of the seven PC patients, four had a family history of cancer.

Linear modeling identified 4556 cytosine CpGs that were significantly (FDR‐adjusted *p*‐value < 0.05) differentially methylated in PC versus controls. A total of 2805 hypomethylated and 1751 hypermethylated CpG markers were identified in the study with the FDR‐adjusted *p*‐value < 0.05. The CpGs were significantly more likely to be hypo‐ rather than hypermethylated (Fisher's exact test, *p* = 0.03) in PC (Table S2).

### 3.1 Pathway enrichment analysis

**FIGURE 1:**
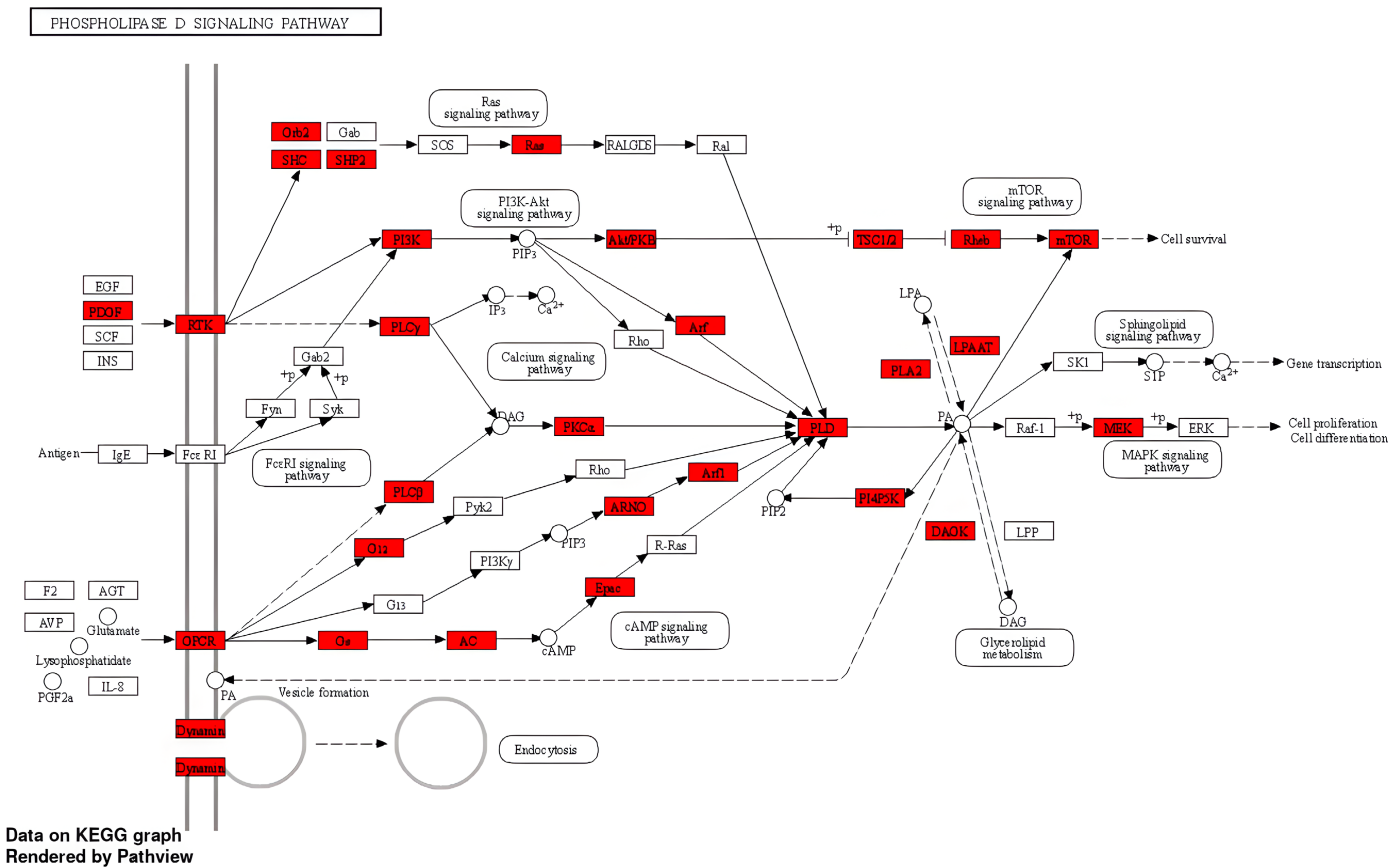
The KEGG pathway analysis: epigenetically altered genes involved in the enriched Phospholipase D signaling.

**FIGURE 2:**
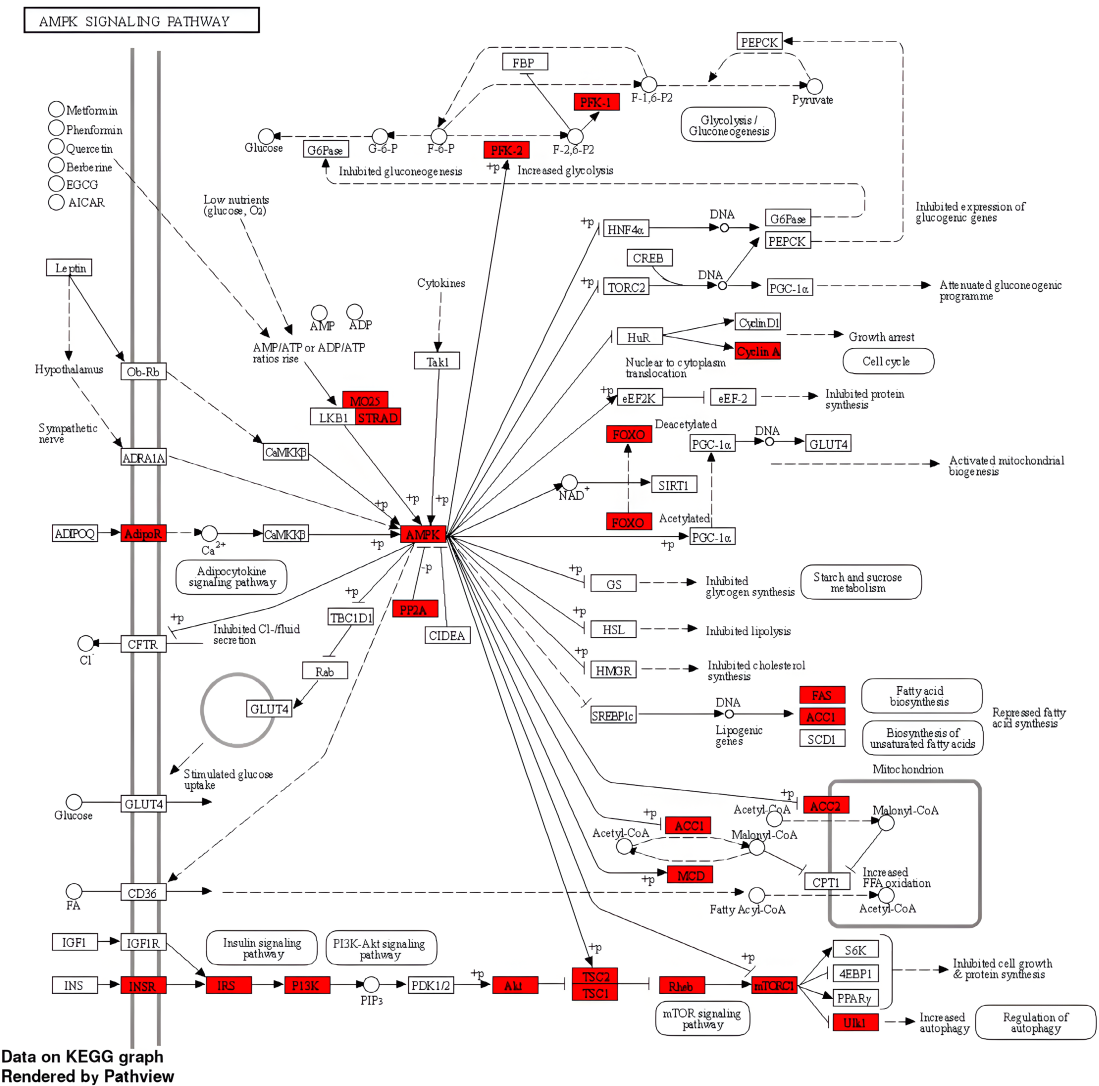
The KEGG pathway analysis: epigenetically altered genes involved in the enriched AMPK signaling pathway.

The pathway enrichment analysis was performed using the significantly differentially methylated genes. We identified 66 significantly altered molecular pathways (FDR *p*‐value < 0.05; Table S3), of which a high percentage appear to be linked to cancer. The top four pathways were: Phospholipase D signaling pathway (Figure 1), AMPK signaling pathway (Figure 2), MAPK signaling pathway (Figure S3), and Notch signaling pathway (Figure S4). Based on current literature, these pathways are significantly linked to cancer and will be individually reviewed in the “Section 4.”

### 3.2 Artificial intelligence for the detection of pancreatic cancer

The predictive accuracy of six separate AI platforms was assessed. In addition, as previously noted, the separate performance of intragenic and extragenic CpG markers was assessed. The AI techniques achieved high model accuracy as represented by the AUC, sensitivity, and specificity values. For example, DL achieved an AUC (95% CI) of 1 (0.95–1) with sensitivity and specificity of 100% for both intragenic and separate intergenic CpG markers. The DL intragenic CpG markers using 10 predictive variables (5‐fold cross‐validation) were: cg16984992, cg16590012, cgg16550438, cg07240877, cg14870958, cg01291513, cg13151361, cg21242417, cg09518293, and cg00019091 (Table 1). Table 2 provides intragenic CpG 10‐marker algorithm based on bootstrapping. All six AI platforms using 10 marker predictors achieved an AUC >0.94. Using 20‐marker algorithms slightly improved the predictive performance with 5‐fold cross‐validation and bootstrapping (Tables 3 and 4). The same was also true for the extragenic (intergenic) CpG markers with 10 and 20 variables and either 5‐fold cross‐validation or bootstrapping (Tables S4 and S5 provide prediction based on 10 variables) and (Tables S6 and S7 provide prediction based on 20 variables). Comparably high diagnostic performances were achieved using more parsimonious 5‐marker algorithms (Tables S8 and S9).

**TABLE 1:** Artificial intelligence and circulating cfDNA in pancreatic cancer: CpG markers located within genes (intragenic; 10‐marker algorithm; 5‐fold cross‐validation).

	SVM	GLM	PAM	RF	LDA	DL
AUC 95% CI	1.0000 (0.9000–1)	1.0000 (0.9000–1)	0.9456 (0.8500–1)	0.9788 (0.9000–1)	1.0000 (0.9000–1)	1.0000 (0.9500–1)
Sensitivity	0.9000	0.9200	0.8500	0.9600	0.9555	1.0000
Specificity	0.9600	0.8800	0.9100	0.9000	0.9700	1.0000

*Note*: Important predictors in order: SVM: cg14224170, cg02805448, cg11148697, cg13339291, cg21466229, cg20727403, cg01430372, cg02403931, cg23178322, cg09632446. GLM: cg22712861, cg13775996, cg08035872, cg06085890, cg26607620, cg18784929, cg10275695, cg06931905, cg02484127, cg18106397. PAM: cg25773935, cg10475689, cg20967889, cg09632446, cg24531698, cg00395140, cg14224170, cg10059324, cg24273843, cg20096979. RF: cg04725405, cg06931905, cg23178322, cg09921548, cg04510815, cg05285759, cg07663722, cg27315239, cg01333650, cg21328081. LDA: cg20967889, cg25773935, cg13339291, cg14224170, cg23178322, cg17897505, cg05065507, cg24877731, cg02610227, cg10845342. DL: cg16984992, cg16590012, cgg16550438, cg07240877, cg14870958, cg01291513, cg13151361, cg21242417, cg09518293, cg00019091.

Abbreviations: DL, Deep Learning; GLM, Generalized Linear Model; LDA, Linear Discriminant Analysis; PAM, Prediction Analysis for Microarrays; RF, Random Forest; SVM, Support Vector Machine.

**TABLE 2:** Artificial intelligence and circulating cfDNA in pancreatic cancer: CpG markers located within genes (intragenic; 10‐marker algorithm; Bootstrapping).

	SVM	GLM	PAM	RF	LDA	DL
AUC 95% CI	1.0000 (0.9000–1)	1.0000 (0.9000–1)	0.9488 (0.8500–1)	0.9800 (0.9000–1)	1.0000 (0.9000–1)	1.0000 (0.9500–1)
Sensitivity	0.9100	0.9200	0.8700	0.9600	0.9566	1.0000
Specificity	0.9600	0.9000	0.9100	0.9100	0.9700	1.0000

*Note*: Important predictors in order: SVM: cg14224170, cg02805448, cg11148697, cg13339291, cg21466229, cg20727403, cg01430372, cg02403931, cg23178322, cg09632446. GLM: cg22712861, cg13775996, cg08035872, cg06085890, cg26607620, cg18784929, cg10275695, cg06931905, cg02484127, cg18106397. PAM: cg25773935, cg10475689, cg20967889, cg09632446, cg24531698, cg00395140, cg14224170, cg10059324, cg24273843, cg20096979. RF: cg04725405, cg06931905, cg23178322, cg09921548, cg04510815, cg05285759, cg07663722, cg27315239, cg01333650, cg21328081. LDA: cg20967889, cg25773935, cg13339291, cg14224170, cg23178322, cg17897505, cg05065507, cg24877731, cg02610227, cg10845342. DL: cg16984992, cg16590012, cgg16550438, cg07240877, cg14870958, cg01291513, cg13151361, cg21242417, cg09518293, cg00019091.

Abbreviations: DL, Deep Learning; GLM, Generalized Linear Model; LDA, Linear Discriminant Analysis; PAM, Prediction Analysis for Microarrays; RF, Random Forest; SVM, Support Vector Machine.

**TABLE 3:** Artificial intelligence and circulating cfDNA in pancreatic cancer: CpG markers located within genes (intragenic; 20‐marker algorithm; 5‐fold cross‐validation).

	SVM	GLM	PAM	RF	LDA	DL
AUC 95% CI	1.0000 (0.9000–1)	1.0000 (0.9000–1)	0.9477 (0.8500–1)	0.9799 (0.9000–1)	1.0000 (0.9000–1)	1.0000 (0.9500–1)
Sensitivity	0.9000	0.9200	0.8600	0.9600	0.9555	1.0000
Specificity	0.9600	0.8800	0.9100	0.9000	0.9700	1.0000

*Note*: Important predictors in order: SVM: cg14224170, cg02805448, cg11148697, cg13339291, cg21466229, cg20727403, cg01430372, cg02403931, cg23178322, cg09632446, cg05065507, cg15127853, cg17897505, cg22486192, cg24877731, cg16984992, cg22873177, cg21506278, cg20967889, cg06900089. GLM: cg22712861, cg13775996, cg08035872, cg06085890, cg26607620, cg18784929, cg10275695, cg06931905, cg02484127, cg18106397, cg21959890, cg17330034, cg09899541, cg02403931, ch.1.1248405R, cg09464192, cg08125081, cg23203809, cg11148697, cg15127853. PAM: cg25773935, cg10475689, cg20967889, cg09632446, cg24531698, cg00395140, cg14224170, cg10059324, cg24273843, cg20096979, cg20727403, cg01430372, cg08940169, cg13339291, cg21466229, cg07240877, cg16590012, cg10299917, cg23499977, cg02403931. RF: cg04725405, cg06931905, cg23178322, cg09921548, cg04510815, cg05285759, cg07663722, cg27315239, cg01333650, cg21328081, cg05729577, cg03407412, cg17570350, cg21669037, cg12525249, cg17498612, cg20203854, cg15306794, cg06823437. LDA: cg20967889, cg25773935, cg13339291, cg14224170, cg23178322, cg17897505, cg05065507, cg24877731, cg02610227, cg10845342, cg22486192, cg02403931, cg10475689, cg08879111, cg01430372, cg09632446, cg16590012, cg22873177, cg02805448, cg15127853. DL: cg16984992, cg16590012, cgg16550438, cg07240877, cg14870958, cg01291513, cg13151361, cg21242417, cg09518293, cg00019091, cg16868591, cg23957525, cg01723761, cg01688293, cg14224638, cg20967889, cg04725405, cg25773935, cg22712861, cg14224170.

Abbreviations: DL, Deep Learning; GLM, Generalized Linear Model; LDA, Linear Discriminant Analysis; PAM, Prediction Analysis for Microarrays; RF, Random Forest; SVM, Support Vector Machine.

**TABLE 4:** Artificial intelligence and circulating cfDNA in pancreatic cancer: CpG markers located within genes (intragenic; 20‐marker algorithm; Bootstrapping).

	SVM	GLM	PAM	RF	LDA	DL
AUC 95% CI	1.0000 (0.9000–1)	1.0000 (0.9000–1)	0.9499 (0.8500–1)	0.9833 (0.9000–1)	1.0000 (0.9000–1)	1.0000 (0.9500–1)
Sensitivity	0.9100	0.9200	0.8900	0.9600	0.9566	1.0000
Specificity	0.9600	0.9000	0.9100	0.9100	0.9700	1.0000

*Note*: Important predictors in order: SVM: cg14224170, cg02805448, cg11148697, cg13339291, cg21466229, cg20727403, cg01430372, cg02403931, cg23178322, cg09632446, cg05065507, cg15127853, cg17897505, cg22486192, cg24877731, cg16984992, cg22873177, cg21506278, cg20967889, cg06900089. GLM: cg22712861, cg13775996, cg08035872, cg06085890, cg26607620, cg18784929, cg10275695, cg06931905, cg02484127, cg18106397, cg21959890, cg17330034, cg09899541, cg02403931, ch.1.1248405R, cg09464192, cg08125081, cg23203809, cg11148697, cg15127853. PAM: cg25773935, cg10475689, cg20967889, cg09632446, cg24531698, cg00395140, cg14224170, cg10059324, cg24273843, cg20096979, cg20727403, cg01430372, cg08940169, cg13339291, cg21466229, cg07240877, cg16590012, cg10299917, cg23499977, cg02403931. RF: cg04725405, cg06931905, cg23178322, cg09921548, cg04510815, cg05285759, cg07663722, cg27315239, cg01333650, cg21328081, cg05729577, cg03407412, cg17570350, cg21669037, cg12525249, cg17498612, cg20203854, cg15306794, cg06823437. LDA: cg20967889, cg25773935, cg13339291, cg14224170, cg23178322, cg17897505, cg05065507, cg24877731, cg02610227, cg10845342, cg22486192, cg02403931, cg10475689, cg08879111, cg01430372, cg09632446, cg16590012, cg22873177, cg02805448, cg15127853. DL: cg16984992, cg16590012, cgg16550438, cg07240877, cg14870958, cg01291513, cg13151361, cg21242417, cg09518293, cg00019091, cg16868591, cg23957525, cg01723761, cg01688293, cg14224638, cg20967889, cg04725405, cg25773935, cg22712861, cg14224170.

Abbreviations: DL, Deep Learning; GLM, Generalized Linear Model; LDA, Linear Discriminant Analysis; PAM, Prediction Analysis for Microarrays; RF, Random Forest; SVM, Support Vector Machine.

### 3.3 Partial least squares discriminant analysis plot and logistic regression models for the detection of pancreatic cancer

The PLS‐DA plot showed good separation between the PC and control groups (Figure S5). The R2 (0.90) and Q2 values (0.81) indicate good classification accuracy and strong predictive relevance of the model (Figure S6). Similarly, the heatmap showed good visual discrimination of PC and control groups based on CpG methylation studies (Figure S7).

The literature indicates that AI is superior to regression analysis for group discrimination.^25^ However, at this time, regression analysis remains a more widely used tool. We therefore also performed logistic regression analysis to develop multi‐biomarker prediction for PC. Using a combination of top five CpG markers (cg19388016, cg04545708, cg13069535, cg17350349, and cg18923221), an AUC (95% CI) = 1.0 (1.0–1.0) with 100% sensitivity and 100% specificity for PC detection was achieved following 10‐fold cross‐validation.

## 4 DISCUSSION

The integration of systems biology including epigenomics, genomics, and transcriptomics has advanced our understanding of the mechanisms of pancreatic cancer. Further, targeted therapy based on these molecular alterations appears to be associated with an improved prognosis.^26^ This further validates the wisdom of the precision medicine approach to pancreatic cancer detection and treatment. DNA methylation plays a crucial role in controlling gene expression and holds promise in the field of cancer biology and ultimately for delivering clinical benefits due to its early role in neoplastic transformation.^27^, ^28^ It is clear from several studies that treatments such as chemotherapy and radiation therapy can induce methylation differences in patients.^29^, ^30^ We therefore focused on methylation changes in circulating cfDNA from the treatment‐naïve patients in this study.

The late presentation and diagnosis of PC remain a fundamental challenge to improving survival statistics.^31^, ^32^ Imaging techniques such as CT scans and MRI which are currently used tools for aiding diagnosis, are useful in the later stages of PC. Also, given their expense, they are not suitable as screening tools. Based on a minimally invasive approach grounded in the principles of precision oncology, we used a combination of circulating cfDNA, genome‐wide epigenetic analysis, and AI to accurately detect PC. For example, using 20‐marker predictive algorithms (Table 4), all six AI platforms achieved an AUC ≥ 0.94, with four achieving an AUC (95% CI) = 1.0 (0.95–1.00). As an example, DL achieved AUC (95% CI) = 1.0 (0.95–1.00), with sensitivity and specificity both at 100%. Similar performance was found with either intragenic markers (Tables 3 and 4) or intergenic (extragenic) markers (Tables S6 and S7). Parsimonious 5‐marker models achieved comparably high diagnostic performances (Tables S8 and S9). Using a non‐AI approach, each CPG marker and top logistic regression model yielded similarly high diagnostic performance.

Recent studies have begun to evaluate the utility of cfDNA for pancreatic cancer detection. Henriksen et al.,^33^ targeted a handful of specific genes. Others, based on meta‐analysis by the Visser et al.,^34^ have used cfDNA obtained from pancreatic juice. For example, using a pooled diagnostic performance strategy, *NPTX2* gene methylation achieved sensitivity of 42% and specificity of 98% for PC detection. However, the need to collect pancreatic juice, which is not easily accessible, would preclude the use of this approach in the routine screening of at‐risk individuals. As noted above, our parsimonious algorithm based on five markers and using DL achieved an AUC (95% CI) = 1 (0.95–1) with a sensitivity and specificity of 100% when either intragenic or intergenic CpGs by themselves were used (Tables S8 and S9, respectively). The study by Ying et al.^35^ analyzed plasma cfDNA using the HumanMethylation450K BeadChip Ilumina kit and assessed the effectiveness of a 4‐panel gene approach. They achieved an AUC of 0.94, a sensitivity of 100%, and a specificity of 90%. Li et al.^36^ performed whole genome methylation analysis using the methylated DNA immunoprecipitation sequencing (MeDIP‐Seq) method on plasma cfDNA but validated the results using a public database in which tissue samples were analyzed with the HumanMethylation450K array. When the methylation markers identified from cfDNA were evaluated in that tissue database, an AUC of 0.975 with sensitivity of 97.1% and a specificity of 98.0% in the training dataset and an AUC of 0.943 with sensitivity of 93.2% and a specificity of 95.2% in a validation dataset. Huang et al.^37^ also performed genome‐wide methylation analysis using cfDNA‐based methyl‐binding domain sequencing (MBD‐seq) method. However, for the prediction analysis, they evaluated the overlapping methylation changes of cfDNA and primary tumor tissues from public databases. Using a large number of CpGs, the top 100 cancer‐type‐specific “differentially hypermethylated CpG islands (DMCGIs),” they reported an AUC of 0.989. Additionally, TET‐assisted pyridine borane sequencing (TAPS), a direct DNA methylation sequencing method that does not require bisulfite conversion of DNA, has been also evaluated using cfDNA.^38^ That study evaluated prediction based on methylation changes in the promoter regions, and an AUC of 0.98 for PC detection was achieved. Also, when applying the cfDNA TAPs seq data to publicly available databases of tumor tissues, they achieved an AUC of 0.81 for PC detection.^38^ These studies fundamentally support our findings that DNA methylation changes and in particular cfDNA analysis have the potential to be accurate, minimally invasive biomarkers for PC. Consistent with precision medicine principles, we used sophisticated analytic approaches namely AI for disease prediction. Epigenome‐wide analysis generates a large volume of data including putative biomarkers. AI techniques are specifically engineered to handle such big data.^39^ Studies have also shown the superiority of Machine Learning/AI techniques including DL^25^ over conventional statistical analysis for disease detection and prediction of medical outcomes.^40^, ^41^

We assessed the importance of the differentially methylated genes on biological pathways, both to further elucidate the molecular mechanisms of PC and also to determine the biological plausibility of our findings. A high percentage of the 66 epigenetically dysregulated molecular pathways identified (Table S3) was related to cancer.

### 4.1 Signaling pathways

Studies indicate that the regulation of cancer cell survival, proliferation, invasion, and growth is significantly influenced by phospholipases.^42^ The role of Phospholipase D in cancer development and management specifically due to its antiapoptotic effects is therefore an area of interest.^43^ We identified 36 differentially methylated genes that were enriched in the Phospholipase D signaling pathway, indicating a plausible role of this signaling pathway in PC pathogenesis. The MAPK signaling pathway was also found to be enriched along with Phospholipase D signaling pathway. A study previously suggested an interaction between Phospholipase D and p38 MAPK, in which the activation of Phospholipase D is necessary for the activation of p38 MAPK signaling.^44^ Our analysis found that 57 genes in the MAPK signaling pathway underwent significant methylation changes in PC. The AMPK signaling pathway was also found to be overrepresented in PC. The AMPK pathway is known to interact with MAPK signaling and to regulate cellular metabolism, cellular survival, cell differentiation, and proliferation.^45^ We also found that the Notch signaling pathway was overrepresented in PC. Studies show that MAPK is involved in regulating the expression of Notch target genes through a transcription factor and several cofactors controls transcription.^46^ In the current study, we identified several biological pathways that were significantly epigenetically altered in pancreatic cancer, one of which was cellular senescence. This process has a dual impact on cancer cells. On one side, it inhibits cell division and enhances the removal of damaged cells by the immune system, thus preventing tumor development. On the other hand, senescence can contribute to tumor progression and relapse by creating an immune‐suppressing environment.^47^, ^48^ Additionally, we found that the RAS signaling pathway is involved in tumor initiation, invasion, and metastasis.^49^ Another important pathway is the Rap1 signaling pathway, which plays a crucial role in regulating key events related to tumor cell migration, invasion, and metastasis.^50^ Moreover, the calcium signaling pathway was found to be significant, with calcium playing a central role in the migration, invasion, and metastasis of PC cells.^51^ The PI3K‐Akt signaling pathway was also identified, and its frequent activation is well‐established in promoting PC aggressiveness.^52^ Among the pathways directly related to cancer, “pathways in cancer” and “pancreatic cancer” signaling showed the possible involvement of several differentially methylated genes in our study. Overall, given the known relationship of these and multiple other overrepresented pathways with cancer, our findings of epigenetic dysregulation of these CpGs and genes in PC appears to be biologically plausible and confirm the great complexity involved in cancer transformation.

We identified several significantly differentially methylated imprinted genes with potential implications for PC. Among these, genes were TP73, SVOPL, DLGAP2, KCNK9, OSBPL5, H19, KCNQ1, HNF1A, MEG8, RASGRF1, NLRP2, and GNAS. Notably, DLGAP2 inhibition in PC cells led to a reduction in proliferation, invasion, and migration abilities.^53^ We found four hypomethylated CpGs on DLGAP2, suggesting possible gene overexpression, which could help explain the aggressive behavior of PC cells. The GNAS gene, involved in cAMP‐PKA pathways, was found to contribute to tumor cell proliferation.^54^ TP73, a tumor suppressor gene belonging to the p53 family of transcription factors, was identified as crucial for tumor progression due to its interaction with p53.^55^ The H19 gene, encoding H19 imprinted maternally expressed transcript, was associated with abnormal Wnt/β‐catenin signaling pathways in PC.^56^ In our study, OSBPL5 exhibited higher transcriptional expression in PC patients^57^ consistent with our findings showing two hypomethylated markers on this gene. RASGRF1, another imprinted gene, demonstrated fusion with other genes, rendering the cells sensitive to targeting of the RAF‐MEK‐ERK pathway, leading to multiple malignancies, including PC.^58^ All these imprinted genes displayed a strong individual predictive accuracy for PC detection, with each having an AUC > 0.90. Among the remaining imprinted genes, MEG8 was found to contribute the epigenetic changes that lead to the progression in epithelial‐mesenchymal transition in PC cells.^59^ HNF1A, a transcription factor regulating pancreatic differentiation and endocrine pancreas homeostasis, is considered a susceptibility gene for PC.^60^ Although KCNK9 was previously studied in association with breast cancer, our findings indicate its potential relevance to PC as well. This gene encodes a potassium channel, and its overexpression in cell lines promotes tumor formation and confers resistance to hypoxia and serum deprivation.^61^ The discoveries from our study shed light on the intricate roles of imprinted genes in PC and open avenues for further research and therapeutic interventions.

While epigenomic therapeutics offer promise in pancreatic cancer therapy,^62^ effective targeted therapy cannot be realized without extensive mapping of the epigenome and determination of the dysregulated molecular networks associated with specific features of PC, such as neoplastic transformation, clonality, and drug resistance. The CA19‐9 is a commonly used blood‐based biomarker for PC detection. CA19‐9 is an epitope that can be found on various proteins, although the precise number and nature of these carrier proteins have not been determined. Nevertheless, some proteins have been identified, including mucins.^63^, ^64^ Examples include *MUC1* and *MUC5AC*, which are known to be elevated in malignancy. At the same time, CA‐19‐9 was identified in nonmalignant tissues in association with *MUC3* and *MUC6*, while *MUC2* has been detected in patients with less advanced tumors.^65^ Additionally, the CA19‐9 epitope was also found on *MUC4* and *MUC16* in PC.^64^, ^66^ Beyond the mucin family, other proteins such as Apolipoprotein B‐100 (*APOB*), kininogen (*KNG1*), *ARVCF*, and Apolipoprotein E (*APOE*) have also been identified as carriers of the CA19‐9 antigen.^64^ However, it is important to emphasize that the expression of the CA19‐9 antigen requires the presence of the Lewis blood group antigen. Patients who lack the genotypic markers for Lewis blood group antigens will not produce the CA19‐9 antigen, even in the presence of malignancy.^67^ We found significant methylation changes in some of the genes known to express the CA‐19‐9 epitope such as *MUC4*, *MUC6*, and *MUC16*. Our work helps to lay the groundwork for this effort in identifying epigenetically dysregulated loci and molecular pathways associated with pancreatic cancer.

Our study is not without limitations. These include the relatively small sample size. Multiple AI strategies, including choice of platforms as detailed in Data S1 “overfitting and computation time,” were utilized to minimize the risk of overfitting. We also separately employed cross‐validation and bootstrapping analytic approaches. Although we did not have a separate test group, we performed cross‐validation procedures to enhance the generalizability of the results. Larger validation studies are now required to confirm our findings. Despite these limitations, using multiple different AI platforms, different CpG markers, and the evaluation of both intra‐ and extragenic CpG markers yielded consistently high and statistically significant prediction of PC. To further address this issue of overfitting with AI, we also performed non‐AI‐based prediction. Using logistic regression analysis, the diagnostic accuracy achieved was comparably high as that obtained with the use of AI. Ten‐fold cross‐validation was performed to minimize overfitting. The high R2 statistic from the PLS‐DA analysis indicated that the variance between PC and normal controls group was significantly explained by the CpG methylation. In addition, the high R2 value indicated that the model has predictive relevance. While the AUCs consistently achieved statistical significance for the models across different AI platforms and with the use of logistic regression‐based analysis, we recommend cautious interpretation of our findings given the small sample size of our study. The risk of a chance finding increases with decreasing sample size.

The ultimate objective of precision oncology is the development of targeted, patient‐specific treatments. The fundamental challenge with current cancer therapy is the clonality and heterogenetic of tumor cells resulting in initial suppression, but subsequent resurgence of resistant clones leading to drug resistance, and clinical recurrence. There is now good evidence that epigenetic modification plays a critical role in tumor cell heterogeneity.^68^ This awareness has stimulated the design of epigenetic based inhibitors to be used in combination with chemotherapy for reprogramming resistant tumors.^69^ Thus, our work of mapping the PC epigenome has the future potential of contributing to the development of targeted therapy.

In summary, using a minimally invasive approach based on epigenome‐wide analysis of circulating cfDNA, consistently high PC prediction was achieved. Further, our analysis found that the genes and molecular pathways that were epigenetically altered are known or suspected to be involved in various aspects of cancer biology giving further biological plausibility to our findings. Larger studies to validate our findings are clearly indicated.

## AUTHOR CONTRIBUTIONS

**Ray O. Bahado‐Singh:** Conceptualization (equal); data curation (equal); funding acquisition (lead); investigation (equal); project administration (equal); resources (equal); supervision (equal); writing – original draft (equal). **Onur Turkoglu:** Data curation (equal); formal analysis (equal); software (equal); writing – review and editing (equal). **Buket Aydas:** Data curation (equal); formal analysis (equal); methodology (equal); software (equal); writing – review and editing (equal). **Sangeetha Vishweswaraiah:** Conceptualization (equal); data curation (equal); investigation (equal); methodology (equal); project administration (equal); software (equal); supervision (equal); validation (equal); visualization (equal); writing – original draft (equal).

## CONFLICT OF INTEREST STATEMENT

These data have partly contributed to a provisional patent application by the home institution, Beaumont Research Institute, for cancer detection.

## Supporting information


Figure S1.

Figure S2.

Figure S3.

Figure S4.

Figure S5.

Figure S6.

Figure S7.

Table S1.

Table S2.

Table S3.

Tables S4–S9.

Data S1.

## Data Availability

Data relevant to the published article will be available from the corresponding author upon request.
