# Supplementary figures and images for: Precision oncology: Artificial intelligence, circulating cell‐free DNA, and the minimally invasive detection of pancreatic cancer—A pilot study
Source: Cancer Med. 2023 Oct 3;12(19):19644–55. doi: 10.1002/cam4.6604 (PMC10587955; doi:10.1002/cam4.6604)

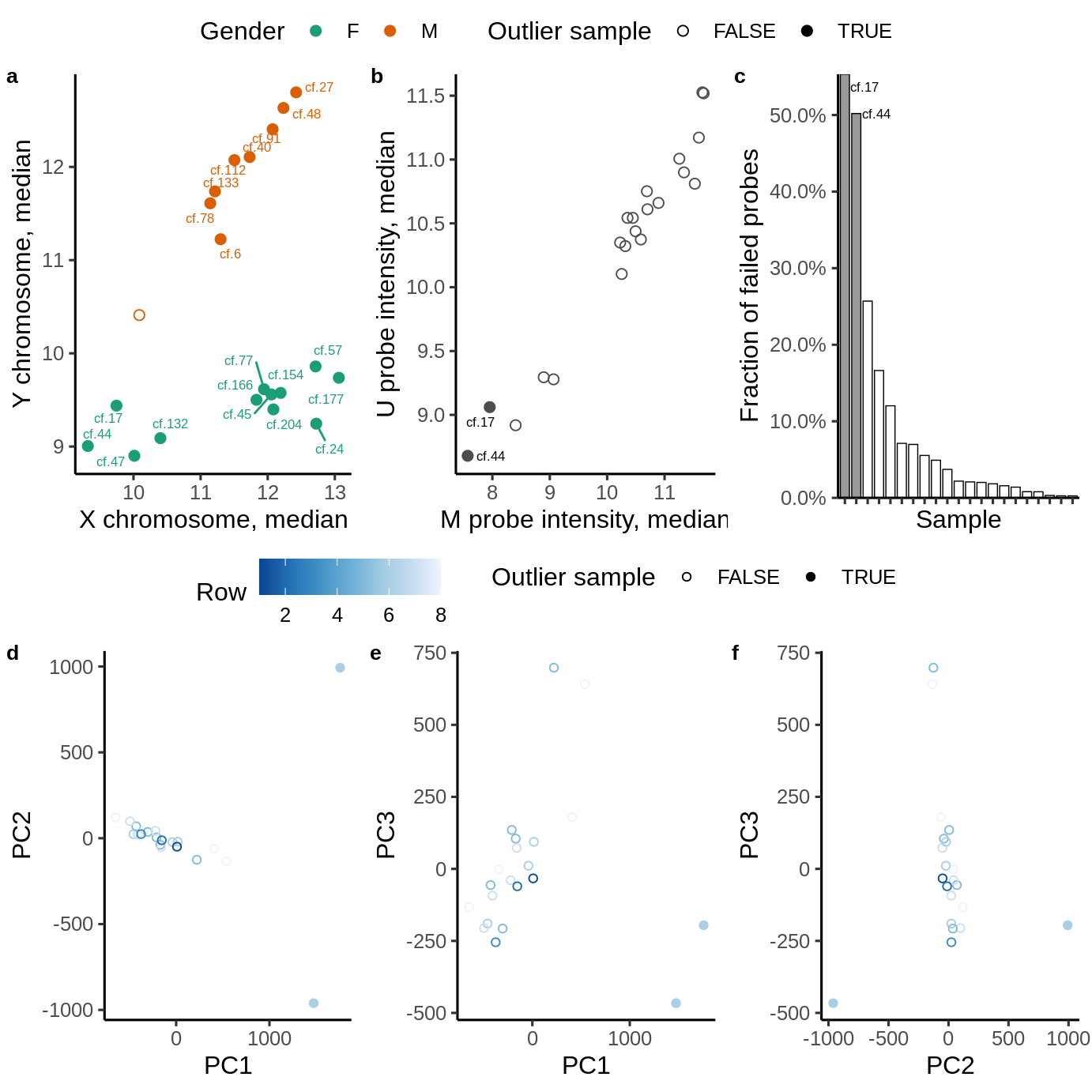

Supplement: Supplementary file 1 — Figure S1. [file CAM4-12-19644-s006.png]

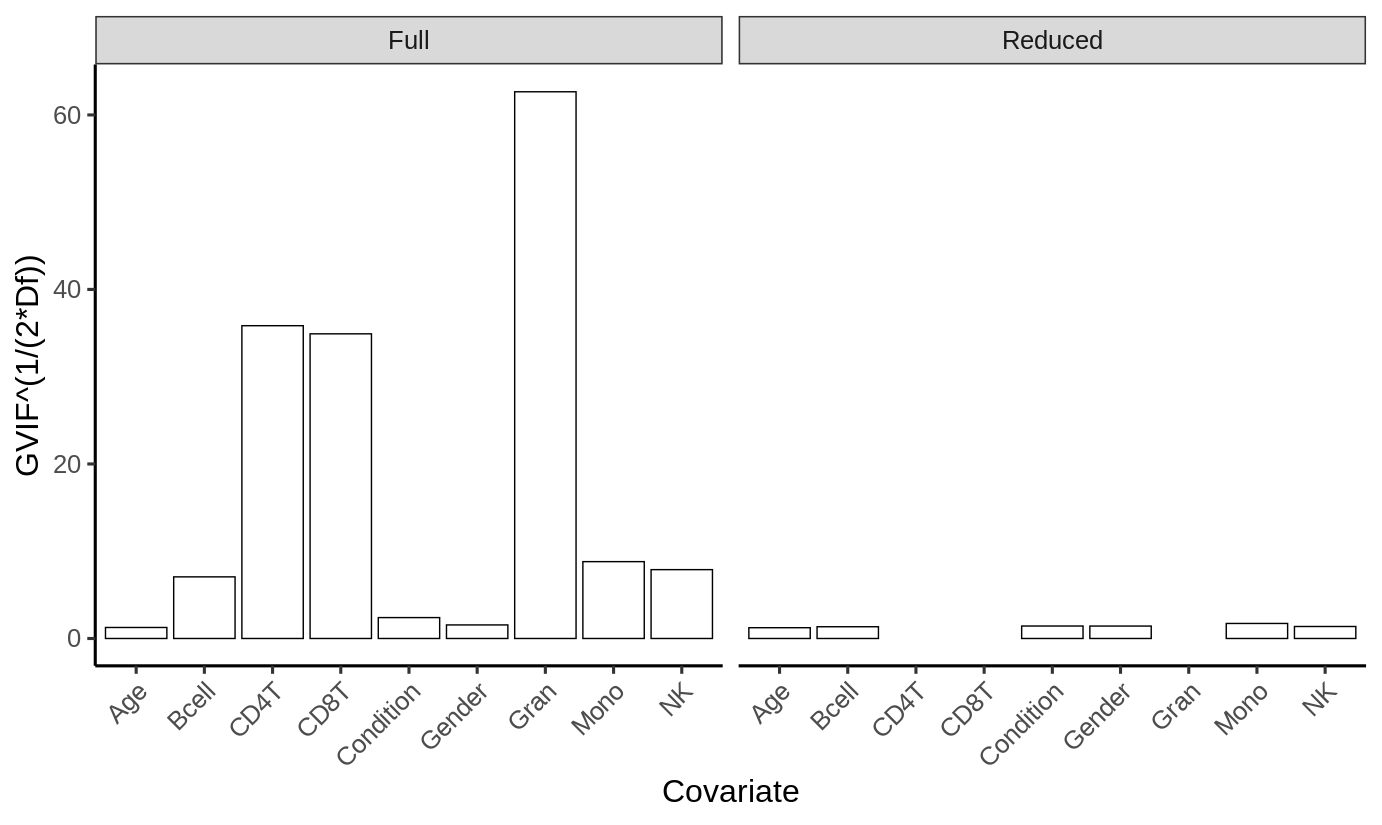

Supplement: Supplementary file 2 — Figure S2. [file CAM4-12-19644-s010.png]

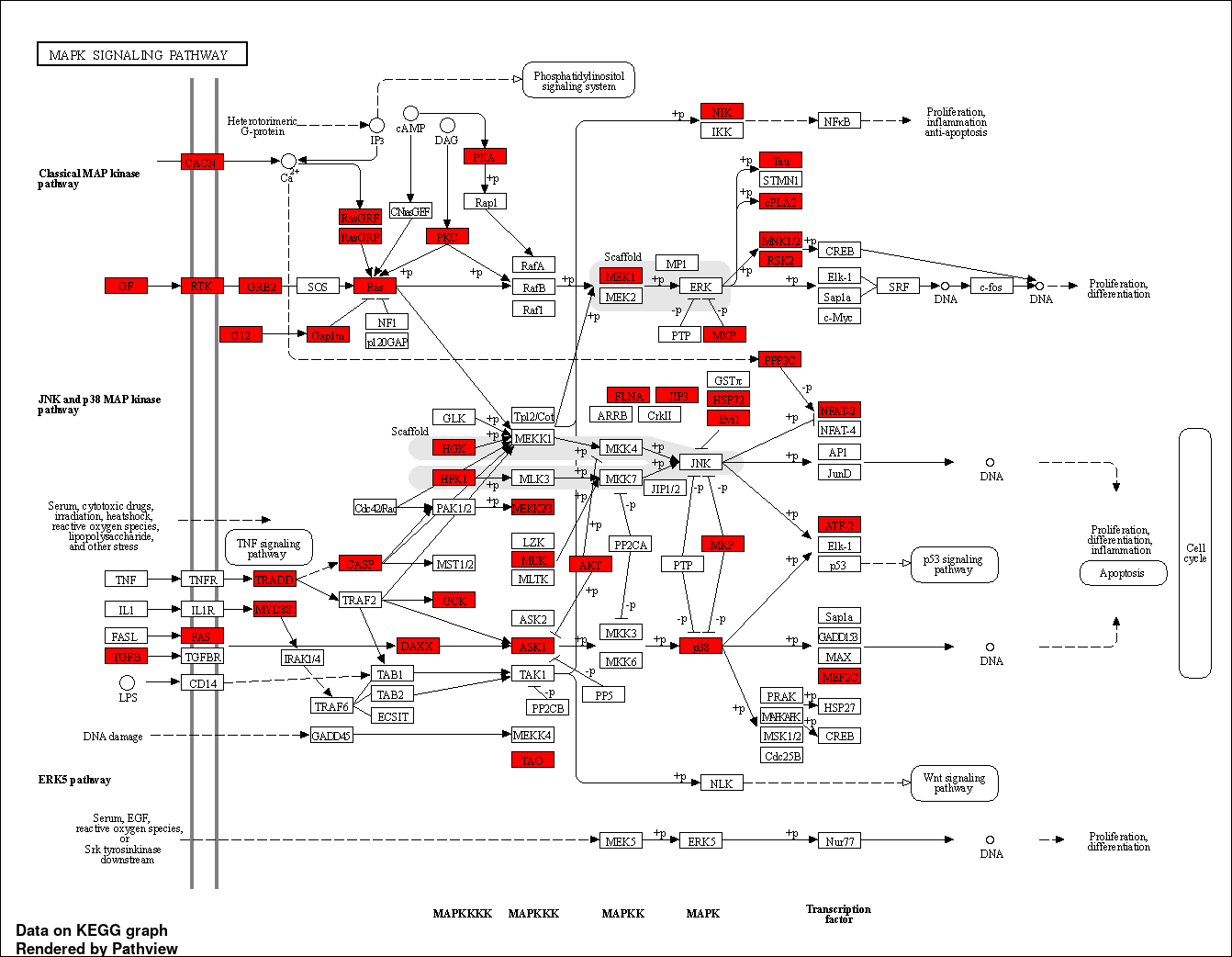

Supplement: Supplementary file 3 — Figure S3. [file CAM4-12-19644-s004.png]

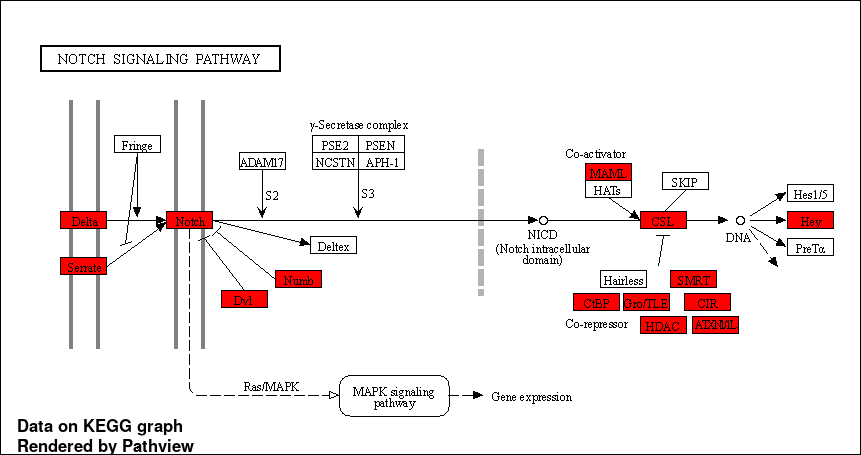

Supplement: Supplementary file 4 — Figure S4. [file CAM4-12-19644-s009.png]

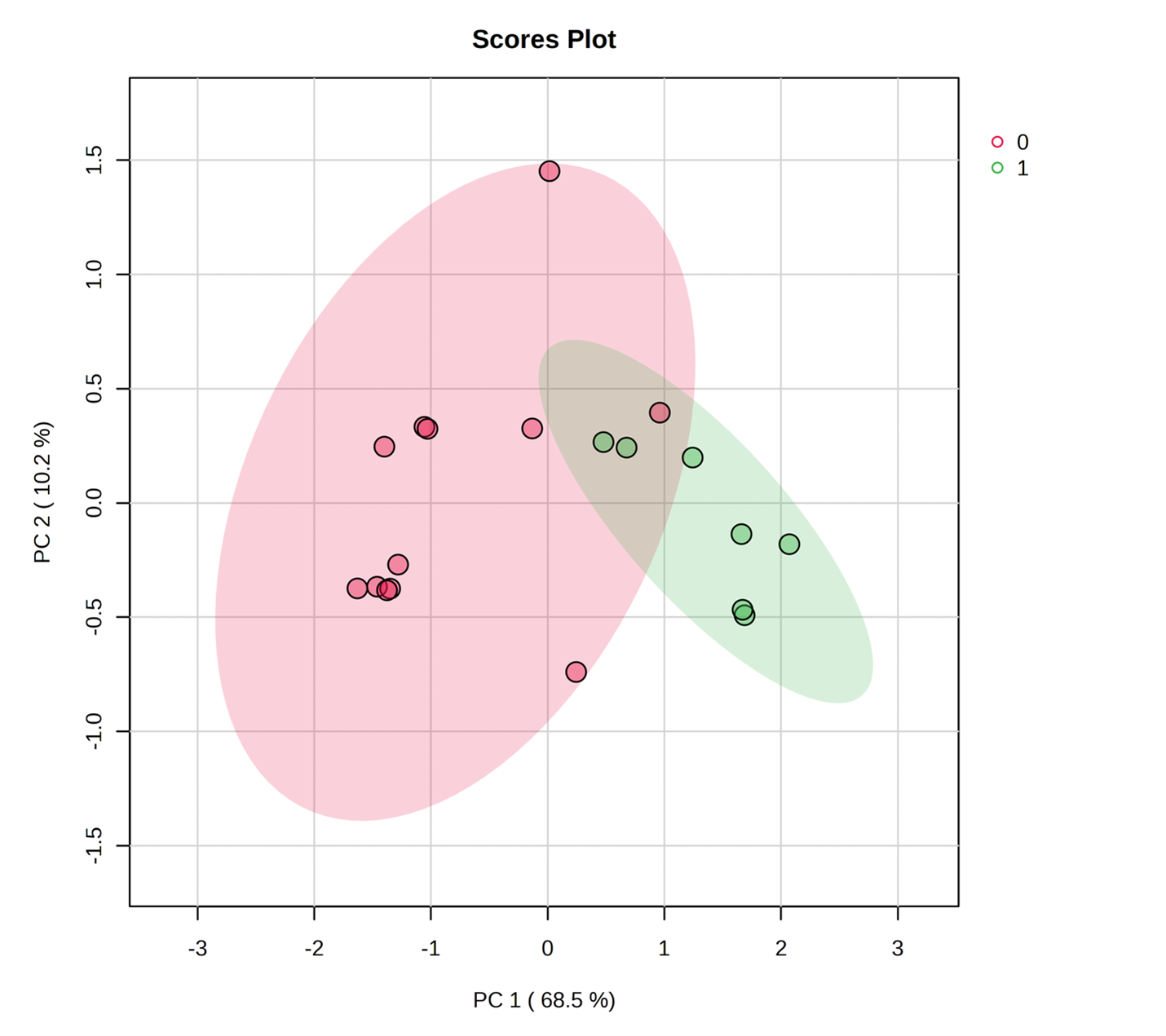

Supplement: Supplementary file 5 — Figure S5. [file CAM4-12-19644-s001.png]

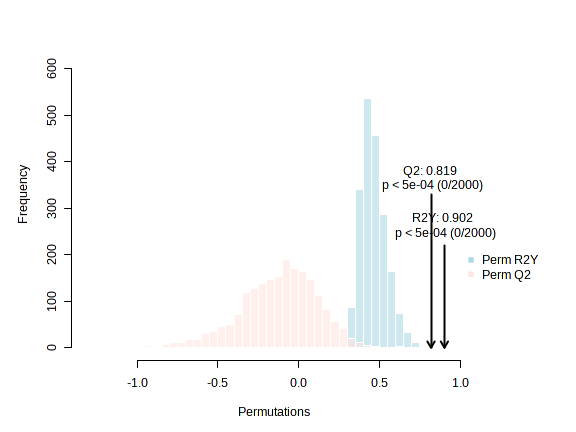

Supplement: Supplementary file 6 — Figure S6. [file CAM4-12-19644-s002.png]

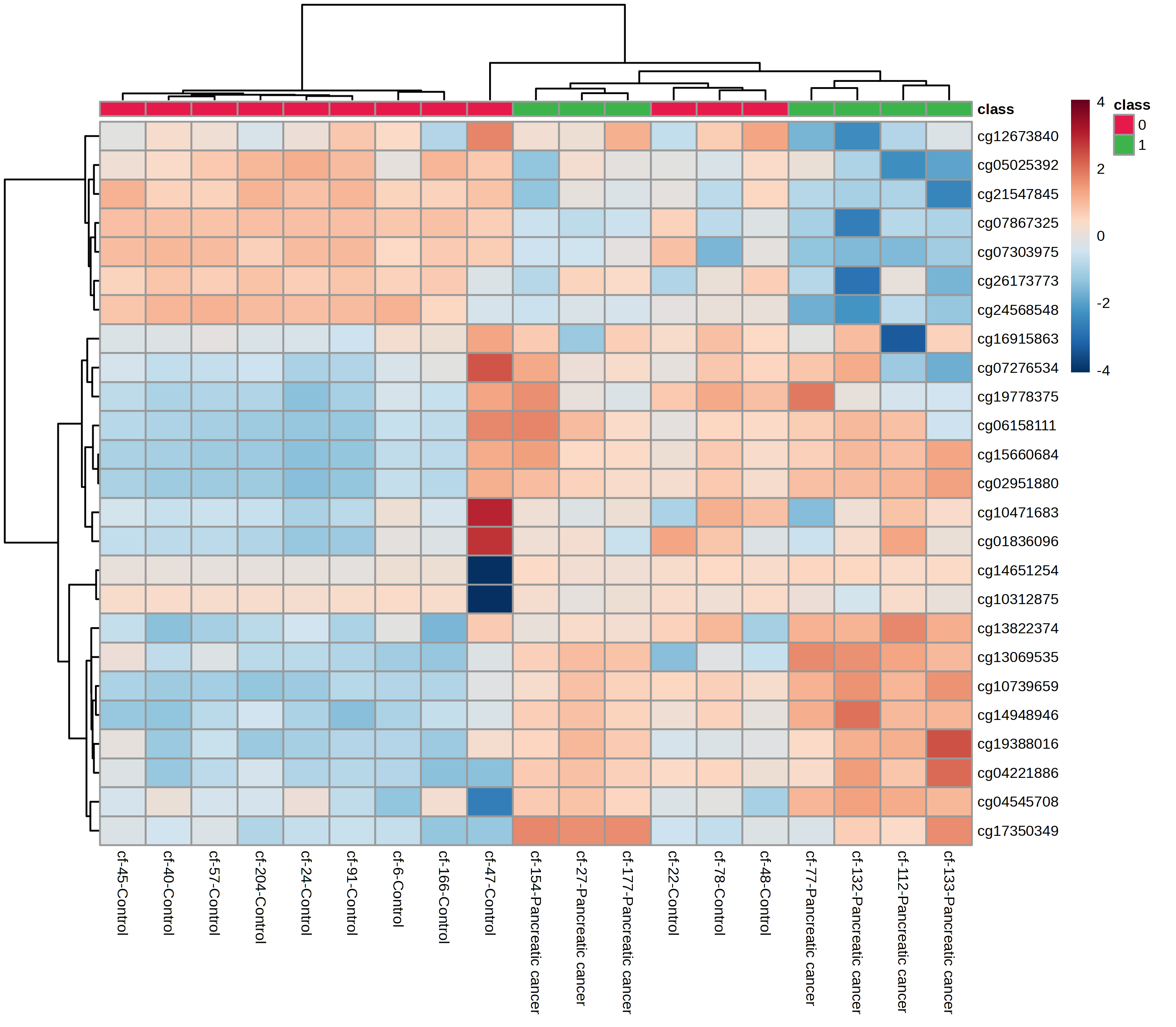

Supplement: Supplementary file 7 — Figure S7. [file CAM4-12-19644-s007.png]
