# Supplementary material for: Precision oncology: Artificial intelligence, circulating cell‐free DNA, and the minimally invasive detection of pancreatic cancer—A pilot study
Source: Cancer Med. 2023 Oct 3;12(19):19644–55. doi: 10.1002/cam4.6604 (PMC10587955; doi:10.1002/cam4.6604)
Supplement: Supplementary file 8 — Table S1. [file CAM4-12-19644-s003.docx]

**Supplemental Table 1:** Demographic and clinical characteristics of pancreatic cancer cases versus controls.

| **Parameter** | **Cases** | **Controls** | **p-value** |
| --- | --- | --- | --- |
| Number of patients | 7 | 14 | - |
| Race - Caucasian | 7 | 14 | - |
| Gender  Female  Male | 4  3 | 8  6 | 0.43 (T) |
| Age - Mean (Standard deviation) | 64.57 (12.95) | 71.71.8 (5.3) | 0.43 (T) |
| BMI - Mean (Standard deviation) | 25.76 (4.97) | 26.81 (5.92) | 0.48 (T) |
| **Histological type and stages of pancreatic cancer study subjects** | | | |

| **Histological type** | **Stage** |
| --- | --- |
| Poorly differentiated adenocarcinoma pancreatic body | NA |
| Adenocarcinoma pancreas head | NA |
| Adenocarcinoma pancreas neck | NA |
| IPMN (pancreatic mass) | NA |
| Pancreatic ductal adenocarcinoma, moderately differentiated | pT3N2 |
| Pancreatic ductal adenocarcinoma, mets to lymph nodes | ypT3 N2 |
| Ductal adenocarcinoma, moderately differentiated, IPMN | pT2 N2 |

*T – T test
