# Supplementary material for: Precision oncology: Artificial intelligence, circulating cell‐free DNA, and the minimally invasive detection of pancreatic cancer—A pilot study
Source: Cancer Med. 2023 Oct 3;12(19):19644–55. doi: 10.1002/cam4.6604 (PMC10587955; doi:10.1002/cam4.6604)
Supplement: Supplementary file 11 — Tables S4–S9. [file CAM4-12-19644-s012.docx]

**Supplemental Table 4:** Artificial intelligence and circulating cfDNA in Pancreatic Cancer: CpG markers located outside genes (intergenic) (10- marker algorithm) (5-fold cross validation).

|  | SVM | GLM | PAM | RF | LDA | DL |
| --- | --- | --- | --- | --- | --- | --- |
| AUC  95% CI | 1.0000  (0.9000-1) | 0.9955  (0.9000-1) | 0.9556  (0.8500-1) | 0.9888  (0.9000-1) | 1.0000  (0.9000-1) | 1.0000  (0.9500-1) |
| Sensitivity | 0.9100 | 0.9200 | 0.8500 | 0.9600 | 0.9555 | 1.0000 |
| Specificity | 0.9300 | 0.8800 | 0.9100 | 0.9000 | 0.9700 | 1.0000 |

Important predictors in order:

SVM: cg03550773, cg14185604, cg01643444, cg27652249, cg19153883, cg26689710, cg00655656, cg27662284, cg17678740, cg08084911

GLM: cg09688773, cg20149137, cg06977823, cg22713460, cg26689710, cg06105068, ch.6.1795380F, cg17678740, ch.1.207470500F, ch.6.19775382R

PAM: cg03969515, cg06959657, cg17678740, cg08084911, cg03721045, cg27652249, cg06977823, cg09688773, cg21749456, cg06105068

RF: cg06940716, cg02532528, cg22082780, cg15708219, cg08084911, cg15313990, cg21592087, cg24824745, cg25980682, cg17979445

LDA: cg03550773, cg22082780, cg26689710, cg09688773, cg14185604, cg03969515, cg01753315, cg27652249, cg13242160, cg06105068

DL: cg03449513, cg26689710, cg14050247, cg10825481, cg22345349, cg22272911, cg22900519, cg01386294, cg03586128, cg08836199

**Supplemental Table 5:** Artificial intelligence and circulating cfDNA in Pancreatic Cancer: CpG markers located outside genes (intergenic) (10 - marker algorithm) (Bootstrapping).

|  | SVM | GLM | PAM | RF | LDA | DL |
| --- | --- | --- | --- | --- | --- | --- |
| AUC  95% CI | 1.0000  (0.9000-1) | 0.9966  (0.9000-1) | 0.9577  (0.8700-1) | 0.9899  (0.9000-1) | 1.0000  (0.9000-1) | 1.0000  (0.9500-1) |
| Sensitivity | 0.9200 | 0.9300 | 0.8800 | 0.9600 | 0.9500 | 1.0000 |
| Specificity | 0.9300 | 0.8900 | 0.9100 | 0.9100 | 0.9800 | 1.0000 |

Important predictors in order:

SVM: cg03550773, cg14185604, cg01643444, cg27652249, cg19153883, cg26689710, cg00655656, cg27662284, cg17678740, cg08084911

GLM: cg09688773, cg20149137, cg06977823, cg22713460, cg26689710, cg06105068, ch.6.1795380F, cg17678740, ch.1.207470500F, ch.6.19775382R

PAM: cg03969515, cg06959657, cg17678740, cg08084911, cg03721045, cg27652249, cg06977823, cg09688773, cg21749456, cg06105068

RF: cg06940716, cg02532528, cg22082780, cg15708219, cg08084911, cg15313990, cg21592087, cg24824745, cg25980682, cg17979445

LDA: cg03550773, cg22082780, cg26689710, cg09688773, cg14185604, cg03969515, cg01753315, cg27652249, cg13242160, cg06105068

DL: cg03449513, cg26689710, cg14050247, cg10825481, cg22345349, cg22272911, cg22900519, cg01386294, cg03586128, cg08836199

**Supplemental Table 6:** Artificial intelligence and circulating cfDNA in Pancreatic Cancer: CpG markers located outside genes (intergenic) (20 - marker algorithm) (5-fold cross validation).

|  | SVM | GLM | PAM | RF | LDA | DL |
| --- | --- | --- | --- | --- | --- | --- |
| AUC  95% CI | 1.0000  (0.9000-1) | 0.9955  (0.9000-1) | 0.9556  (0.8500-1) | 0.9888  (0.9000-1) | 1.0000  (0.9000-1) | 1.0000  (0.9500-1) |
| Sensitivity | 0.9100 | 0.9200 | 0.8500 | 0.9600 | 0.9555 | 1.0000 |
| Specificity | 0.9300 | 0.8800 | 0.9100 | 0.9000 | 0.9700 | 1.0000 |

Important predictors in order:

SVM: cg03550773, cg14185604, cg01643444, cg27652249, cg19153883, cg26689710, cg00655656, cg27662284, cg17678740, cg08084911, cg12749087, cg23999337, cg06977823, cg11483359, cg11786916, cg09688773, cg16901465, cg06105068, cg14050247, cg10086080

GLM: cg09688773, cg20149137, cg06977823, cg22713460, cg26689710, cg06105068, ch.6.1795380F, cg17678740, ch.1.207470500F, ch.6.19775382R, cg21749456, cg00655656, cg22082780, cg03550773, cg01731848, cg16901465, cg13615109, cg06959657, cg15748376, cg23936531

PAM: cg03969515, cg06959657, cg17678740, cg08084911, cg03721045, cg27652249, cg06977823, cg09688773, cg21749456, cg06105068, cg03550773, cg23999337, cg26689710, cg16901465, cg12749087, cg00895680, cg01386294, cg01381374, cg14050247, cg04611493

RF: cg06940716, cg02532528, cg22082780, cg15708219, cg08084911, cg15313990, cg21592087, cg24824745, cg25980682, cg17979445, cg01559375, cg17077180, cg26689710, ch.1.207470500F, cg01902036, cg27561567, cg09762920, cg18854706, cg18953829, cg18697996

LDA: cg03550773, cg22082780, cg26689710, cg09688773, cg14185604, cg03969515, cg01753315, cg27652249, cg13242160, cg06105068, cg16964373, cg27662284, cg00655656, cg06959657, cg06977823, cg04611493, cg22345349, cg10825481, cg08084911, cg14867639

DL: cg03449513, cg26689710, cg14050247, cg10825481, cg22345349, cg22272911, cg22900519, cg01386294, cg03586128, cg08836199, cg16132851, cg14455590, cg05993665, cg16901465, cg00895680, cg03550773, cg06940716, cg03969515, cg09688773, cg14185604

**Supplemental Table 7:** Artificial intelligence and circulating cfDNA in Pancreatic Cancer: CpG markers located outside genes (intergenic) (20 - marker algorithm) (Bootstrapping).

|  | SVM | GLM | PAM | RF | LDA | DL |
| --- | --- | --- | --- | --- | --- | --- |
| AUC  95% CI | 1.0000  (0.9000-1) | 0.9966  (0.9000-1) | 0.9577  (0.8700-1) | 0.9899  (0.9000-1) | 1.0000  (0.9000-1) | 1.0000  (0.9500-1) |
| Sensitivity | 0.9200 | 0.9300 | 0.8800 | 0.9600 | 0.9500 | 1.0000 |
| Specificity | 0.9300 | 0.8900 | 0.9100 | 0.9100 | 0.9800 | 1.0000 |

Important predictors in order:

SVM: cg03550773, cg14185604, cg01643444, cg27652249, cg19153883, cg26689710, cg00655656, cg27662284, cg17678740, cg08084911, cg12749087, cg23999337, cg06977823, cg11483359, cg11786916, cg09688773, cg16901465, cg06105068, cg14050247, cg10086080

GLM: cg09688773, cg20149137, cg06977823, cg22713460, cg26689710, cg06105068, ch.6.1795380F, cg17678740, ch.1.207470500F, ch.6.19775382R, cg21749456, cg00655656, cg22082780, cg03550773, cg01731848, cg16901465, cg13615109, cg06959657, cg15748376, cg23936531

PAM: cg03969515, cg06959657, cg17678740, cg08084911, cg03721045, cg27652249, cg06977823, cg09688773, cg21749456, cg06105068, cg03550773, cg23999337, cg26689710, cg16901465, cg12749087, cg00895680, cg01386294, cg01381374, cg14050247, cg04611493

RF: cg06940716, cg02532528, cg22082780, cg15708219, cg08084911, cg15313990, cg21592087, cg24824745, cg25980682, cg17979445, cg01559375, cg17077180, cg26689710, ch.1.207470500F, cg01902036, cg27561567, cg09762920, cg18854706, cg18953829, cg18697996

LDA: cg03550773, cg22082780, cg26689710, cg09688773, cg14185604, cg03969515, cg01753315, cg27652249, cg13242160, cg06105068, cg16964373, cg27662284, cg00655656, cg06959657, cg06977823, cg04611493, cg22345349, cg10825481, cg08084911, cg14867639

DL: cg03449513, cg26689710, cg14050247, cg10825481, cg22345349, cg22272911, cg22900519, cg01386294, cg03586128, cg08836199, cg16132851, cg14455590, cg05993665, cg16901465, cg00895680, cg03550773, cg06940716, cg03969515, cg09688773, cg14185604

**Supplemental Table 8:** Artificial intelligence and circulating cfDNA in Pancreatic Cancer: CpG markers located within genes (intragenic) (5 - marker algorithm) (5-fold cross validation).

|  | SVM | GLM | PAM | RF | LDA | DL |
| --- | --- | --- | --- | --- | --- | --- |
| AUC  95% CI | 0.9988  (0.9000-1) | 0.9900  (0.9000-1) | 0.9277  (0.8500-1) | 0.9575  (0.9000-1) | 0.9988  (0.9000-1) | 1.0000  (0.9500-1) |
| Sensitivity | 0.8900 | 0.9000 | 0.8000 | 0.9500 | 0.9333 | 1.0000 |
| Specificity | 0.9500 | 0.8500 | 0.9000 | 0.8500 | 0.9500 | 1.0000 |

Important predictors in order:

SVM: cg14224170, cg02805448, cg11148697, cg13339291, cg21466229

GLM: cg22712861, cg13775996, cg08035872, cg06085890, cg26607620

PAM: cg25773935, cg10475689, cg20967889, cg09632446, cg24531698

RF: cg04725405, cg06931905, cg23178322, cg09921548, cg04510815

LDA: cg20967889, cg25773935, cg13339291, cg14224170, cg23178322

DL: cg16984992, cg16590012, cgg16550438, cg07240877, cg14870958

**Supplemental Table 9:** Artificial intelligence and circulating cfDNA in Pancreatic Cancer: CpG markers located outside genes (intergenic) (5 - marker algorithm) (5-fold cross validation).

|  | SVM | GLM | PAM | RF | LDA | DL |
| --- | --- | --- | --- | --- | --- | --- |
| AUC  95% CI | 0.9888  (0.9000-1) | 0.9855  (0.9000-1) | 0.9377  (0.8500-1) | 0.9675  (0.9000-1) | 0.9788  (0.9000-1) | 1.0000  (0.9500-1) |
| Sensitivity | 0.8700 | 0.9000 | 0.8000 | 0.9500 | 0.9333 | 1.0000 |
| Specificity | 0.9300 | 0.8500 | 0.9000 | 0.8500 | 0.9500 | 1.0000 |

Important predictors in order:

SVM: cg03550773, cg14185604, cg01643444, cg27652249, cg19153883

GLM: cg09688773, cg20149137, cg06977823, cg22713460, cg26689710

PAM: cg03969515, cg06959657, cg17678740, cg08084911, cg03721045

RF: cg06940716, cg02532528, cg22082780, cg15708219, cg08084911

LDA: cg03550773, cg22082780, cg26689710, cg09688773, cg14185604

DL: cg03449513, cg26689710, cg14050247, cg10825481, cg22345349
