# Supplementary material for: Precision oncology: Artificial intelligence, circulating cell‐free DNA, and the minimally invasive detection of pancreatic cancer—A pilot study
Source: Cancer Med. 2023 Oct 3;12(19):19644–55. doi: 10.1002/cam4.6604 (PMC10587955; doi:10.1002/cam4.6604)
Supplement: Supplementary file 12 — Data S1. [file CAM4-12-19644-s011.docx]

**Supplementary Artificial Intelligence (AI) Methods**

**Artificial Intelligence analysis Method Data Preprocessing.** The logged value of each CpG was centered by its beta values and auto scaled by its standard deviation (s) to regulate the balance between high and low-intensity features, and to moderate the heteroscedasticity. We utilized quantile normalization to diminish sample-to-sample difference.

**Deep Learning.** Classical machine learning techniques make predictions directly from a set of features that have been pre-specified by the user. However, representation learning techniques transform features into some intermediate representation prior to mapping them to final predictions. DL is a form of representation learning that uses multiple transformation steps to create very complex features.

DL is categorized into feed-forward artificial neural networks (ANNs), with multiple layers between the input and output layers. DL finds the correct mathematical manipulation to turn the input into the output, whether the relationship is linear or non-linear. The network propagates through the layers while calculating the probability of each output. At first, the DL creates a map of virtual neurons and assigns random numerical values, or "weights", to connections between them. The weights and inputs are multiplied and yield an output value between ‘0’ and ‘1’. If the network does not recognize a particular pattern, an algorithm adjusts the weights. That way the algorithm can make certain parameters more influential, until it determines the correct mathematical manipulation to fully process the data.

In the DL model, more than one hidden layer (y) connect the input (x) and output layer (z) via a weight (W) matrix. We calculated the activation value of the hidden layer (y) by the sigmoid of the multiplication of the input sample ‘x ‘with the weight matrix value ‘W’ and bias ‘b’. The transpose of the weight matrix W and the bias b were then used to construct the output (z) layer. The optimal set of the weight matrix W and bias b are chosen in order to minimize the difference between the input layer (x) and the output layer (z).

To start, the first hidden layer (y) was activated by providing the sample input (x) to the first layer and deciding on the best parameters (W, b). Then, the second layer was predicted by utilizing first hidden layer (y). The same process was repeated for all remaining layers-updating the weights and bias for each layer. Subsequently, we used back-propagation to regulate the parameters for all hidden layers. Finally, softmax classifier was used for the final hidden layer to assign new labels to the samples. We used the h2o R computer package to tune the parameters of the DL model.

**Other Machine Learning Algorithms.** In addition to DL we also evaluated a representative set of five ML algorithms which have been applied to metabolomics and genomics data for classification and regression analyses.

**Below is a synopsis of each of these ML approaches:**

**Random Forest (RF)** is a widely used machine learning algorithm based on decision tree theory. It works well for high-dimensional data and can accommodate unbalanced and missing values in the data set.

**Support vector machine (SVM)** is another machine learning algorithm that has been used to separate Over-represented canonical pathways, biological processes and molecular processes was identified. Over-represented canonical pathways, biological processes and molecular processes was identified.

**Linear Discriminant Analysis (LDA)** is closely related to analysis of variance (ANOVA) and regression analysis and is used to express one dependent variable as a linear combination of other features or measurements.

**Prediction Analysis for Microarrays (PAM)** has been used to perform sample classification from gene expression data.

**Generalized linear Model (GLM) -** (Logistic Regression) - measures the relationship between the categorical dependent variable and one or more independent variables by estimating probabilities using a logistic function, which is the cumulative logistic distribution.

To get the optimal predictive performance, we used the caret R computer package ^1^ to tune the parameters in the models.

**Modeling & Evaluation:**

Two-step validation was utilized for these analyses. There were two different data sets, first was utilized to build the model and test it, the second one was used to validate the model.

While using the two-step validation method, two different techniques were utilized to find out the best model and calculate the performance metrics: 10-fold Cross validation and Bootstrapping.

1. **5-fold Cross Validation:** The first data set was split into a training to train the model first with a portion of the data and a test group (remaining portion) on which the performance of the developed model is then determined. Here we randomly divided the available set of samples into two parts: a training set and a test or hold-out set. The model was fit on the training set, and the fitted model was used to predict the responses for the observations in the hold-out set. Estimates were used to select best model, and to give an idea of the test error of the final chosen model. Idea was to randomly divide the data into 5 equal-sized parts. We left out part 5, fit the model to the other 4 parts (combined), and then obtained predictions for the left-out 5th part. This was done in turn for each part k = 1, 2...5, and then the results were combined. This process was repeated a total of ten times and the average AUC, sensitivity, specificity and 95% confidence intervals for the test set were calculated. Then, as the validation step, AUC, sensitivity, specificity and 95% confidence intervals for the validation data set were calculated, too.
2. **Bootstrapping:** The bootstrap is a flexible and powerful statistical tool that allowed us to use a computer to mimic the process of obtaining new data sets, so that we were able to estimate the variability of our estimate without generating additional samples. Rather than repeatedly obtaining independent data sets from the population, we instead obtained distinct data sets by repeatedly sampling observations from the original data set with replacement. Each of these “bootstrap data sets” was created by sampling with replacement and was the same size as our original dataset. As a result, some observations appeared more than once in each bootstrap data set and some not at all. To estimate prediction error using the bootstrap, we used each bootstrap dataset as our training sample, and the original sample as our test sample. This process was repeated a total of ten times and the average AUC, sensitivity, specificity and 95% confidence intervals for the test set were calculated. Then, as the validation step, AUC, sensitivity, specificity and 95% confidence intervals for the validation data set were calculated, too.

The following parameters were used to tune the DL model:

• Epochs (number of passes of the full training set),

• l1 (penalty to converge the weights of the model to 0),

• l2 (penalty to prevent the enlargement of the weights),

• Input dropout ratio (ratio of ignored neurons in the input layer during training),

• Number of hidden layers;

The parameters that were used to tune the SVM model was the cost of classification; to tune the RF model was the number of trees to fit; to tune the PAM model was the threshold amount for shrinking toward the centroid.

**Overfitting and computation time.** Two common challenges with the use of DL are overfitting and the computation time. DL is prone to overfitting because of the added layers of abstraction, which allows it to model rare dependencies in the training data. To avoid overfitting in the DL model, we used three regularization parameters: L1, which increases model stability and causes many weights to become 0 and L2, which prevents weight enlargement. L1 lets only strong weights survive (constant pulling force towards zero), while L2 prevents any single weight from getting too. Dropout ^2^ has recently been introduced as a powerful generalization technique, and is available as a parameter per layer, including the input layer. The key idea is to randomly drop units (along with their connections) from the neural network during training. This prevents units from excessive co-adapting. The third parameter that we used for avoiding overfitting in DL model was the input dropout ratio which controls the amount of input layer neurons that are randomly dropped (set to zero) and controls overfitting with respect to the input data. This is particularly useful for high-dimensional noisy data.

**Feature Importance.** Feature (predictor) importance was estimated using a model-based approach. A feature is considered important if it contributes to the model performance. We used the variable importance functions in h2o (varimp) and in caret R packages (varimp) to rank the models features in each of the predictive algorithms.

**References:**

1. Kuhn M. Building Predictive Models in R Using the caret Package. . Journal of Statistical Software. 2008;28: 1-26.

2. Srivastava N, Hinton G, Krizhevsky A, Sutskever I, Salakhutdinov R. Dropout: a simple way to prevent neural networks from overfitting. J. Mach. Learn. Res. 2014;15: 1929-1958.
